# Supplementary material for: Unveiling the Bioactive Potential of the Invasive Jellyfish Phyllorhiza punctata Through Integrative Transcriptomic and Proteomic Analyses
Source: Biomolecules. 2025 Aug 4;15(8):1121. doi: 10.3390/biom15081121 (PMC12383608; doi:10.3390/biom15081121)
Supplement: Supplementary file 1 [file biomolecules-15-01121-s001.zip › Suplementary Material/FileS3/PEPFOLD4/XP_065052450_5840/clusters.html]

 PEP-FOLD clusters


Download archive of all models  here   

|  |  |  |
| --- | --- | --- |
| # | file | sOPEP |
| # cluster 1 - Size: 2 |  |  |
|  | XP\_065052450\_5840-model1 | -20.9311 |
|  | XP\_065052450\_5840-model1.1 | -16.5735 |
| # cluster 2 - Size: 1 |  |  |
|  | XP\_065052450\_5840-model2 | -20.226 |
| # cluster 3 - Size: 3 |  |  |
|  | XP\_065052450\_5840-model3 | -19.943 |
|  | XP\_065052450\_5840-model3.1 | -19.4479 |
|  | XP\_065052450\_5840-model3.2 | -19.0458 |
| # cluster 4 - Size: 17 |  |  |
|  | XP\_065052450\_5840-model4 | -18.985 |
|  | XP\_065052450\_5840-model4.1 | -18.985 |
|  | XP\_065052450\_5840-model4.2 | -18.902 |
|  | XP\_065052450\_5840-model4.3 | -18.902 |
|  | XP\_065052450\_5840-model4.4 | -18.9006 |
|  | XP\_065052450\_5840-model4.5 | -18.9006 |
|  | XP\_065052450\_5840-model4.6 | -18.6973 |
|  | XP\_065052450\_5840-model4.7 | -18.5301 |
|  | XP\_065052450\_5840-model4.8 | -18.0392 |
|  | XP\_065052450\_5840-model4.9 | -17.8825 |
|  | XP\_065052450\_5840-model4.10 | -17.6518 |
|  | XP\_065052450\_5840-model4.11 | -17.574 |
|  | XP\_065052450\_5840-model4.12 | -17.043 |
|  | XP\_065052450\_5840-model4.13 | -16.5687 |
|  | XP\_065052450\_5840-model4.14 | -16.4592 |
|  | XP\_065052450\_5840-model4.15 | -16.1886 |
|  | XP\_065052450\_5840-model4.16 | -15.7822 |
| # cluster 5 - Size: 73 |  |  |
|  | XP\_065052450\_5840-model5 | -18.3708 |
|  | XP\_065052450\_5840-model5.1 | -18.3572 |
|  | XP\_065052450\_5840-model5.2 | -18.3572 |
|  | XP\_065052450\_5840-model5.3 | -18.3572 |
|  | XP\_065052450\_5840-model5.4 | -18.2277 |
|  | XP\_065052450\_5840-model5.5 | -18.2277 |
|  | XP\_065052450\_5840-model5.6 | -18.2277 |
|  | XP\_065052450\_5840-model5.7 | -18.2277 |
|  | XP\_065052450\_5840-model5.8 | -18.2272 |
|  | XP\_065052450\_5840-model5.9 | -18.2272 |
|  | XP\_065052450\_5840-model5.10 | -18.2272 |
|  | XP\_065052450\_5840-model5.11 | -18.2272 |
|  | XP\_065052450\_5840-model5.12 | -18.2272 |
|  | XP\_065052450\_5840-model5.13 | -18.2272 |
|  | XP\_065052450\_5840-model5.14 | -18.2272 |
|  | XP\_065052450\_5840-model5.15 | -18.2272 |
|  | XP\_065052450\_5840-model5.16 | -18.2272 |
|  | XP\_065052450\_5840-model5.17 | -18.2272 |
|  | XP\_065052450\_5840-model5.18 | -18.2272 |
|  | XP\_065052450\_5840-model5.19 | -18.227 |
|  | XP\_065052450\_5840-model5.20 | -18.227 |
|  | XP\_065052450\_5840-model5.21 | -18.2229 |
|  | XP\_065052450\_5840-model5.22 | -18.2042 |
|  | XP\_065052450\_5840-model5.23 | -18.2042 |
|  | XP\_065052450\_5840-model5.24 | -18.2042 |
|  | XP\_065052450\_5840-model5.25 | -18.1928 |
|  | XP\_065052450\_5840-model5.26 | -18.1802 |
|  | XP\_065052450\_5840-model5.27 | -18.114 |
|  | XP\_065052450\_5840-model5.28 | -18.114 |
|  | XP\_065052450\_5840-model5.29 | -18.114 |
|  | XP\_065052450\_5840-model5.30 | -18.114 |
|  | XP\_065052450\_5840-model5.31 | -18.114 |
|  | XP\_065052450\_5840-model5.32 | -18.114 |
|  | XP\_065052450\_5840-model5.33 | -18.114 |
|  | XP\_065052450\_5840-model5.34 | -18.1029 |
|  | XP\_065052450\_5840-model5.35 | -18.0904 |
|  | XP\_065052450\_5840-model5.36 | -18.0904 |
|  | XP\_065052450\_5840-model5.37 | -18.0857 |
|  | XP\_065052450\_5840-model5.38 | -18.0707 |
|  | XP\_065052450\_5840-model5.39 | -18.0496 |
|  | XP\_065052450\_5840-model5.40 | -18.0496 |
|  | XP\_065052450\_5840-model5.41 | -18.0462 |
|  | XP\_065052450\_5840-model5.42 | -18.0443 |
|  | XP\_065052450\_5840-model5.43 | -18.0443 |
|  | XP\_065052450\_5840-model5.44 | -18.0443 |
|  | XP\_065052450\_5840-model5.45 | -18.0443 |
|  | XP\_065052450\_5840-model5.46 | -18.0359 |
|  | XP\_065052450\_5840-model5.47 | -18.0197 |
|  | XP\_065052450\_5840-model5.48 | -18.0197 |
|  | XP\_065052450\_5840-model5.49 | -18.0093 |
|  | XP\_065052450\_5840-model5.50 | -18.0093 |
|  | XP\_065052450\_5840-model5.51 | -18.0093 |
|  | XP\_065052450\_5840-model5.52 | -17.9848 |
|  | XP\_065052450\_5840-model5.53 | -17.9797 |
|  | XP\_065052450\_5840-model5.54 | -17.9793 |
|  | XP\_065052450\_5840-model5.55 | -17.9511 |
|  | XP\_065052450\_5840-model5.56 | -17.9497 |
|  | XP\_065052450\_5840-model5.57 | -17.9369 |
|  | XP\_065052450\_5840-model5.58 | -17.9369 |
|  | XP\_065052450\_5840-model5.59 | -17.8875 |
|  | XP\_065052450\_5840-model5.60 | -17.8547 |
|  | XP\_065052450\_5840-model5.61 | -17.8475 |
|  | XP\_065052450\_5840-model5.62 | -17.7897 |
|  | XP\_065052450\_5840-model5.63 | -17.7309 |
|  | XP\_065052450\_5840-model5.64 | -17.7309 |
|  | XP\_065052450\_5840-model5.65 | -17.6816 |
|  | XP\_065052450\_5840-model5.66 | -17.605 |
|  | XP\_065052450\_5840-model5.67 | -17.5246 |
|  | XP\_065052450\_5840-model5.68 | -17.4496 |
|  | XP\_065052450\_5840-model5.69 | -17.2654 |
|  | XP\_065052450\_5840-model5.70 | -17.1066 |
|  | XP\_065052450\_5840-model5.71 | -16.9436 |
|  | XP\_065052450\_5840-model5.72 | -14.1076 |
| # cluster 6 - Size: 1 |  |  |
|  | XP\_065052450\_5840-model6 | -17.0112 |
| # cluster 7 - Size: 1 |  |  |
|  | XP\_065052450\_5840-model7 | -16.2716 |
| # cluster 8 - Size: 1 |  |  |
|  | XP\_065052450\_5840-model8 | -15.1445 |
| # cluster 9 - Size: 1 |  |  |
|  | XP\_065052450\_5840-model9 | -14.7197 |
|  |  |  |
